# Supplementary material for: HIVprotI: an integrated web based platform for prediction and design of HIV proteins inhibitors
Source: J Cheminform. 2018 Mar 9;10:12. doi: 10.1186/s13321-018-0266-y (PMC5845081; doi:10.1186/s13321-018-0266-y)
Supplement: Supplementary file 2 — Additional file 2. Source code of HIVProtI web server. [file 13321_2018_266_MOESM2_ESM.zip › HIVprotI_Source-code/hivprotI/weka-3-6-6/doc/weka/gui/arffviewer/ArffTableCellRenderer.html]

ArffTableCellRenderer


---


|  |  |  |
| --- | --- | --- |
| **Overview** \| **Package** \| **Class** \| **Tree** \| **Deprecated** \| **Index** \| **Help** \| **Weka's home** | |  |
| **PREV CLASS**   **NEXT CLASS** | **FRAMES**    **NO FRAMES**     **All Classes** |  |
| SUMMARY: NESTED \| FIELD \| CONSTR \| METHOD | DETAIL: FIELD \| CONSTR \| METHOD |  |


---


## weka.gui.arffviewer Class ArffTableCellRenderer

```
java.lang.Object
  java.awt.Component
      java.awt.Container
          javax.swing.JComponent
              javax.swing.JLabel
                  javax.swing.table.DefaultTableCellRenderer
                      weka.gui.arffviewer.ArffTableCellRenderer
```

**All Implemented Interfaces:**: java.awt.image.ImageObserver, java.awt.MenuContainer, java.io.Serializable, javax.accessibility.Accessible, javax.swing.SwingConstants, javax.swing.table.TableCellRenderer

---

``` public class ArffTableCellRenderer extends javax.swing.table.DefaultTableCellRenderer ```

Handles the background colors for missing values differently than the
DefaultTableCellRenderer.

**Version:**
:   $Revision: 7059 $

**Author:**
:   FracPete (fracpete at waikato dot ac dot nz)

**See Also:**: Serialized Form

---

**Nested Class Summary**

**Nested classes/interfaces inherited from class javax.swing.table.DefaultTableCellRenderer**

`javax.swing.table.DefaultTableCellRenderer.UIResource`

**Nested classes/interfaces inherited from class javax.swing.JComponent**

`javax.swing.JComponent.AccessibleJComponent`

**Nested classes/interfaces inherited from class java.awt.Component**

`java.awt.Component.BaselineResizeBehavior`


**Field Summary**

**Fields inherited from class javax.swing.JComponent**

`TOOL_TIP_TEXT_KEY, UNDEFINED_CONDITION, WHEN_ANCESTOR_OF_FOCUSED_COMPONENT, WHEN_FOCUSED, WHEN_IN_FOCUSED_WINDOW`

**Fields inherited from class java.awt.Component**

`BOTTOM_ALIGNMENT, CENTER_ALIGNMENT, LEFT_ALIGNMENT, RIGHT_ALIGNMENT, TOP_ALIGNMENT`

**Fields inherited from interface javax.swing.SwingConstants**

`BOTTOM, CENTER, EAST, HORIZONTAL, LEADING, LEFT, NEXT, NORTH, NORTH_EAST, NORTH_WEST, PREVIOUS, RIGHT, SOUTH, SOUTH_EAST, SOUTH_WEST, TOP, TRAILING, VERTICAL, WEST`

**Fields inherited from interface java.awt.image.ImageObserver**

`ABORT, ALLBITS, ERROR, FRAMEBITS, HEIGHT, PROPERTIES, SOMEBITS, WIDTH`


**Constructor Summary**

`ArffTableCellRenderer()`
  
          initializes the Renderer with a standard color

`ArffTableCellRenderer(java.awt.Color missingColor,
java.awt.Color missingColorSelected)`
  
          initializes the Renderer with the given colors

`ArffTableCellRenderer(java.awt.Color missingColor,
java.awt.Color missingColorSelected,
java.awt.Color highlightColor,
java.awt.Color highlightColorSelected)`
  
          initializes the Renderer with the given colors


| **Method Summary** | |
| --- | --- |
| `java.awt.Component` | `getTableCellRendererComponent(javax.swing.JTable table, java.lang.Object value, boolean isSelected, boolean hasFocus, int row, int column)`             Returns the default table cell renderer. |

**Methods inherited from class javax.swing.table.DefaultTableCellRenderer**

`firePropertyChange, invalidate, isOpaque, repaint, repaint, repaint, revalidate, setBackground, setForeground, updateUI, validate`

**Methods inherited from class javax.swing.JLabel**

`getAccessibleContext, getDisabledIcon, getDisplayedMnemonic, getDisplayedMnemonicIndex, getHorizontalAlignment, getHorizontalTextPosition, getIcon, getIconTextGap, getLabelFor, getText, getUI, getUIClassID, getVerticalAlignment, getVerticalTextPosition, imageUpdate, setDisabledIcon, setDisplayedMnemonic, setDisplayedMnemonic, setDisplayedMnemonicIndex, setHorizontalAlignment, setHorizontalTextPosition, setIcon, setIconTextGap, setLabelFor, setText, setUI, setVerticalAlignment, setVerticalTextPosition`

**Methods inherited from class javax.swing.JComponent**

`addAncestorListener, addNotify, addVetoableChangeListener, computeVisibleRect, contains, createToolTip, disable, enable, firePropertyChange, firePropertyChange, getActionForKeyStroke, getActionMap, getAlignmentX, getAlignmentY, getAncestorListeners, getAutoscrolls, getBaseline, getBaselineResizeBehavior, getBorder, getBounds, getClientProperty, getComponentPopupMenu, getConditionForKeyStroke, getDebugGraphicsOptions, getDefaultLocale, getFontMetrics, getGraphics, getHeight, getInheritsPopupMenu, getInputMap, getInputMap, getInputVerifier, getInsets, getInsets, getListeners, getLocation, getMaximumSize, getMinimumSize, getNextFocusableComponent, getPopupLocation, getPreferredSize, getRegisteredKeyStrokes, getRootPane, getSize, getToolTipLocation, getToolTipText, getToolTipText, getTopLevelAncestor, getTransferHandler, getVerifyInputWhenFocusTarget, getVetoableChangeListeners, getVisibleRect, getWidth, getX, getY, grabFocus, isDoubleBuffered, isLightweightComponent, isManagingFocus, isOptimizedDrawingEnabled, isPaintingForPrint, isPaintingTile, isRequestFocusEnabled, isValidateRoot, paint, paintImmediately, paintImmediately, print, printAll, putClientProperty, registerKeyboardAction, registerKeyboardAction, removeAncestorListener, removeNotify, removeVetoableChangeListener, requestDefaultFocus, requestFocus, requestFocus, requestFocusInWindow, resetKeyboardActions, reshape, scrollRectToVisible, setActionMap, setAlignmentX, setAlignmentY, setAutoscrolls, setBorder, setComponentPopupMenu, setDebugGraphicsOptions, setDefaultLocale, setDoubleBuffered, setEnabled, setFocusTraversalKeys, setFont, setInheritsPopupMenu, setInputMap, setInputVerifier, setMaximumSize, setMinimumSize, setNextFocusableComponent, setOpaque, setPreferredSize, setRequestFocusEnabled, setToolTipText, setTransferHandler, setVerifyInputWhenFocusTarget, setVisible, unregisterKeyboardAction, update`

**Methods inherited from class java.awt.Container**

`add, add, add, add, add, addContainerListener, addPropertyChangeListener, addPropertyChangeListener, applyComponentOrientation, areFocusTraversalKeysSet, countComponents, deliverEvent, doLayout, findComponentAt, findComponentAt, getComponent, getComponentAt, getComponentAt, getComponentCount, getComponents, getComponentZOrder, getContainerListeners, getFocusTraversalKeys, getFocusTraversalPolicy, getLayout, getMousePosition, insets, isAncestorOf, isFocusCycleRoot, isFocusCycleRoot, isFocusTraversalPolicyProvider, isFocusTraversalPolicySet, layout, list, list, locate, minimumSize, paintComponents, preferredSize, printComponents, remove, remove, removeAll, removeContainerListener, setComponentZOrder, setFocusCycleRoot, setFocusTraversalPolicy, setFocusTraversalPolicyProvider, setLayout, transferFocusBackward, transferFocusDownCycle`

**Methods inherited from class java.awt.Component**

`action, add, addComponentListener, addFocusListener, addHierarchyBoundsListener, addHierarchyListener, addInputMethodListener, addKeyListener, addMouseListener, addMouseMotionListener, addMouseWheelListener, bounds, checkImage, checkImage, contains, createImage, createImage, createVolatileImage, createVolatileImage, dispatchEvent, enable, enableInputMethods, firePropertyChange, firePropertyChange, firePropertyChange, firePropertyChange, firePropertyChange, getBackground, getBounds, getColorModel, getComponentListeners, getComponentOrientation, getCursor, getDropTarget, getFocusCycleRootAncestor, getFocusListeners, getFocusTraversalKeysEnabled, getFont, getForeground, getGraphicsConfiguration, getHierarchyBoundsListeners, getHierarchyListeners, getIgnoreRepaint, getInputContext, getInputMethodListeners, getInputMethodRequests, getKeyListeners, getLocale, getLocation, getLocationOnScreen, getMouseListeners, getMouseMotionListeners, getMousePosition, getMouseWheelListeners, getName, getParent, getPeer, getPropertyChangeListeners, getPropertyChangeListeners, getSize, getToolkit, getTreeLock, gotFocus, handleEvent, hasFocus, hide, inside, isBackgroundSet, isCursorSet, isDisplayable, isEnabled, isFocusable, isFocusOwner, isFocusTraversable, isFontSet, isForegroundSet, isLightweight, isMaximumSizeSet, isMinimumSizeSet, isPreferredSizeSet, isShowing, isValid, isVisible, keyDown, keyUp, list, list, list, location, lostFocus, mouseDown, mouseDrag, mouseEnter, mouseExit, mouseMove, mouseUp, move, nextFocus, paintAll, postEvent, prepareImage, prepareImage, remove, removeComponentListener, removeFocusListener, removeHierarchyBoundsListener, removeHierarchyListener, removeInputMethodListener, removeKeyListener, removeMouseListener, removeMouseMotionListener, removeMouseWheelListener, removePropertyChangeListener, removePropertyChangeListener, repaint, repaint, resize, resize, setBounds, setBounds, setComponentOrientation, setCursor, setDropTarget, setFocusable, setFocusTraversalKeysEnabled, setIgnoreRepaint, setLocale, setLocation, setLocation, setName, setSize, setSize, show, show, size, toString, transferFocus, transferFocusUpCycle`

**Methods inherited from class java.lang.Object**

`equals, getClass, hashCode, notify, notifyAll, wait, wait, wait`

**Constructor Detail**

### ArffTableCellRenderer

```
public ArffTableCellRenderer()
```

:   initializes the Renderer with a standard color

---


### ArffTableCellRenderer

```
public ArffTableCellRenderer(java.awt.Color missingColor,
                             java.awt.Color missingColorSelected)
```

:   initializes the Renderer with the given colors

    **Parameters:**: `missingColor` - the color for missing values: `missingColorSelected` - the color selected missing values

---


### ArffTableCellRenderer

```
public ArffTableCellRenderer(java.awt.Color missingColor,
                             java.awt.Color missingColorSelected,
                             java.awt.Color highlightColor,
                             java.awt.Color highlightColorSelected)
```

:   initializes the Renderer with the given colors

    **Parameters:**: `missingColor` - the color for missing values: `missingColorSelected` - the color selected missing values: `highlightColor` - the color for highlighted values: `highlightColorSelected` - the color selected highlighted values


**Method Detail**

### getTableCellRendererComponent

```
public java.awt.Component getTableCellRendererComponent(javax.swing.JTable table,
                                                        java.lang.Object value,
                                                        boolean isSelected,
                                                        boolean hasFocus,
                                                        int row,
                                                        int column)
```

:   Returns the default table cell renderer.
    stuff for the header is taken from here

    :   **Specified by:**: `getTableCellRendererComponent` in interface `javax.swing.table.TableCellRenderer` **Overrides:**: `getTableCellRendererComponent` in class `javax.swing.table.DefaultTableCellRenderer`
    :   **Parameters:**: `table` - the table this object belongs to: `value` - the actual cell value: `isSelected` - whether the cell is selected: `hasFocus` - whether the cell has the focus: `row` - the row in the table: `column` - the column in the table **Returns:**: the rendering component


---


|  |  |  |
| --- | --- | --- |
| **Overview** \| **Package** \| **Class** \| **Tree** \| **Deprecated** \| **Index** \| **Help** \| **Weka's home** | |  |
| **PREV CLASS**   **NEXT CLASS** | **FRAMES**    **NO FRAMES**     **All Classes** |  |
| SUMMARY: NESTED \| FIELD \| CONSTR \| METHOD | DETAIL: FIELD \| CONSTR \| METHOD |  |


---
